# Phytochemical Analyses, Antioxidant and Anticancer Activities of Ethanolic Leaf Extracts of *Moringa oleifera* Lam. Varieties

**DOI:** 10.3390/plants10112348

**Published:** 2021-10-30

**Authors:** Bilques Farooq, Bhupendra Koul, Deveshi Mahant, Dhananjay Yadav

**Affiliations:** 1 School of Bioengineering and Biosciences, Lovely Professional University, Phagwara 144411, India; bilquesfarooq123@gmail.com (B.F.); deveshi.selina@gmail.com (D.M.); 2 Department of Medical Biotechnology, Yeungnam University, Gyeongsan 38541, Korea

**Keywords:** Jaffna, HPLC, FRAP, moringin, MTT assay, HepG2

## Abstract

*Moringa oleifera* Lam. (Moringaceae) is revered as s ‘miracle tree’ due to its remarkable nutritional, medicinal and industrial uses. In our study, a comparative analysis of the nutritional parameters (antioxidant activity, sugar content—TSS, total soluble proteins—TSP and mineral contents), phytochemicals (HPLC analysis of four anticancer compounds), and cytotoxicity of *M. oleifera* leaf extracts (MLEs) of five selected varieties (conventional, PKM-1, PKM-2, ODC, and Jaffna), was performed. Jaffna variety possessed the highest antioxidant activity (FRAP) followed by other four varieties. The trend observed was: Jaffna (9.47 µg/mL, 18.48 µg/mL, 29.39 µg/mL, and 35.37 µg/mL) > PKM-1 (4.82 µg/mL, 7.63 µg/mL, 22.33 µg/mL, and 27.71 µg/mL) > PKM-2 (2.10 µg/mL, 7.04 µg/mL, 13.18 µg/mL, and 21.78 µg/mL) > ODC (0.17 µg/mL, 2.10 µg/mL, 4.41 µg/mL and 13.94 µg/mL) > Conventional (0.05 µg/mL, 1.08 µg/mL, 2.86 µg/mL, and 5.40 µg/mL), total soluble proteins (TSP) [0.69 ± 0.01 and 0.94 ± 0.01 µg/mL (young and mature stage, respectively)], sugar content (TSS) [0.39 ± 0.01 and 0.51 ± 0.01 µg/mL (young and mature stage, respectively)], chlorophyll content [1.07 ± 0.01 (plantlet stage), 1.36 ± 0.003 (vegetative-stage), 0.82 ± 0.004 (reproductive stage) mg/g], followed by the other four varieties. The trend observed for cytotoxic activities of ethanolic MLEs on HepG2 cell line, based on the IC50 values, was conventional (1.22 mg/mL) > ODC (0.90 mg/mL) > PKM-2 (0.65 mg/mL) > PKM-1 (0.35 mg/mL) > Jaffna (0.15 mg/mL). The results of HPLC quantification of anticancer compounds [beta-sitosterol (0.244%), quercetin (0.216%), kaempferol (0.013%), and moringin (0.063%)] was also in consonance with that of MTT assay. In summary, the trend observed in all the parameters tested was Jaffna > PKM-1 > PKM-2 > ODC > conventional. Thus, Jaffna variety has a better potential to combat malnutrition and cancer and must be recommended for commercial plantations.

## 1. Introduction

*Moringa oleifera* Lam. (Moringaceae) is a ‘miracle tree’ which possesses excellent medicinal and nutritional properties and can combat malnutrition among the young population of the world who are devoid of a nutritious diet [1,2]. It is interesting to note that every part of this tree (leaves, flower, pods, stem-bark, roots, and gum) exhibits one or more nutritional and pharmacological properties. It is a proven superfood that harbors several minerals, vitamins, amino acids, and fatty acids [3,4,5,6,7,8].

The pharmacological properties such as cardiac stimulant, anti-oxidant, anti-inflammatory, anti-bacterial, anti-fungal, anti-pyretic, anti-epileptic, anti-cancer, anti-tumor, anti-ulcer, diuretic, anti-hypertensive, anti-diabetic, anti-ulcer and hepatoprotective can be attributed to the presence of bioactive compounds such as 3-O-(6′-O-oleoyl-β-Dglucopyranosyl)-β-sitosterol, 4-(4′-O-acetyl-α-L-rhamnopyranosyloxy) benzyl isothiocyanate, 4-(α-L-rhamnopyranosyloxy) benzyl glucosinolate, ascorbic acid, isoquercetin, kaempferitrin, Kaempferol, moringine, niazimicin, niaziminin A and B, niazirin, niazirinin, O-ethyl-4-(α-Lrhamnosyloxy) Benzylcarbamate, pterygospermin, quercetin, β-carotene, β-sitosterol, and β-sitosterol-3-O-β-Dglucopyranoside [9,10]. The consumption of *M. oleifera* leaf extracts (MLE) can be beneficial in the treatment of altogether 300 diseases including arthritis, cancer, brain dysfunction, diabetes, hypertension, obesity, skin diseases, etc. [11,12,13]. Moreover, adding Moringa leaf powder to the diet of lactating mothers improves the quality and nutrient content of milk, for the proper growth of the child [14]. Besides nutritional and medicinal properties, it has several industrial applications as well. The leafy biomass is itself used as food and fodder while the MLE has plant growth-enhancing potential [15]. The seed oil is a source of biopesticide, biodiesel, and lubricant and also finds application in cosmetics [16]. Moreover, the seed powder has biosorbent property and can therefore be deployed in water purification regimes [3,9,17,18]. 

In the recent past, the public and the private sectors have shown interest in its commercial plantation due to its exceptional nutritional and therapeutic values, increasing demand for value-added products, potential to be a source of biofuel, climate resilience, and adaptability to new regions. Moringa tree is also a suitable candidate for reforestation program. Its cultivation shall be an alternative source of income for the poor farmers and shall open new vistas for the employment-seeking youth of rural communities [9,16,19].

In our previous study, we performed a comparison of antibacterial and plant growth enhancer property of five (conventional, PKM-1, PKM-2, ODC, and Jaffna) *M. oleifera* varieties [9]. Among them, Jaffna exhibited excellent bacteriostatic and plant growth enhancer properties (Supplementary Tables S1 and S2). The aim of our study was to quantify the amount of protein, sugar, chlorophyll, and bioactive compounds present in leaves of five varieties of *M. oleifera*. The research also focuses on the antioxidant and anticancer activity of *M. oleifera varieties*, so as to suggest the elite variety. Jaffna leaf extract showed a higher amount of protein, sugar, chlorophyll, and bioactive compounds and also performed well in antioxidant and anticancer activities, compared to others. Hence, Jaffna is a promising variety that can be used to treat deficiency diseases and can be deployed in the food and beverage industries. 

## 2. Material and Methods 

### 2.1. Plant Material 

Healthy and uniform seeds of five *Moringa oleifera* Lam. varieties [conventional, PKM-1 (Periyakulam-1), PKM-2 (Periyakulam-2), ODC (Oddanchatram), and Jaffna)], were procured from Tamil Nadu Agriculture University (TAU), India. The wings of the seeds were removed and were soaked in water for 15 min. The seeds were then wrapped in wet paper towels and incubated for sprouting at 25 °C for three days. The seedlings of the aforementioned varieties were transferred into separate plastic pots (3.9 × 3.5 inches) filled with soil and soilrite (soil conditioning mixture) in 1:1 ratio (*w*/*w*) and kept in a polyhouse maintained at 30 °C, relative humidity (RH) of 60%, with a 16/8 h light/dark photoperiod.

### 2.2. Moringa Leaf Extract (MLE) Preparation

Fresh leaves of 3 months and 15 months old plants were harvested, shade dried (5 days), powdered, and sieved through a mesh (pore size: 297 microns). Leaf powder (50 g each) of all the varieties was suspended in conical flasks containing 80% ethanol (100 mL) and incubated on a rotary shaker set at 250 rpm, for 6 h. The filtered MLEs were concentrated at 40–45 °C on a rotary evaporator set at 200 rpm. Thereafter, MLEs (~5 mg/mL) were labeled and refrigerated for further use [20]. MLEs were used for the estimation of total soluble sugars (TSS) and total soluble proteins (TSP).

### 2.3. Estimation of Sugar Content

The sugar content of MLEs of five varieties at two different stages of development (young: 3 months old; mature stage: 15 months old) was estimated by the procedure of DuBois [21]. Phenol (1 mL) and sulphuric acid (5 mL) were added sequentially to the MLE (2 mL each of five different varieties). The test tubes containing the reaction mixture were incubated for 20 min in a water bath set at 30 °C. In a hot acidic medium, glucose becomes dehydrated to hydroxymethylfurfural which, in the presence of phenol, gives a yellow-brown color that can be read and measured spectrophotometrically at 490 nm [22]. Glucose was used to plot the standard curve. 

### 2.4. Estimation of Total Soluble Protein (TSP)

The total soluble protein (TSP) was measured by the procedure of Lowry et al. [23]. The phenolics and pigments were removed by the procedure of Mattoo et al. (1986) [24]. MLEs (200 μL) of each of the five varieties, at young (3-months-old) and mature (15-months-old) growth stage, were taken in test tubes (triplicate) and the volume was adjusted to 1 mL with deionized water. Thereafter, freshly prepared Lowry’s reagent [4% CuSO_4_·5H_2_O (*w*/*v*) + 2% sodium carbonate (*w*/*v*) + 1% SDS (*w*/*v*), 0.4% NaOH (*w*/*v*) and 0.16% sodium potassium tartrate (*w*/*v*)] was added and the reaction mixture was incubated for an hour at RT prior to the addition of the Folin-Ciocalteu reagent (FCR). The absorbance was read at 660 nm spectrophotometrically [25]. BSA was used as a standard protein.

### 2.5. Estimation of Chlorophyll Content

The chlorophyll a, b, and total chlorophyll content was estimated by the IUPAC standardized procedure of Arnon [26]. Fresh leaves (5 g) of *M. oleifera* varieties (5 varieties) were homogenized (80% acetone) and filtered through Whatman filter paper no. 1. The absorbance of the filtrate was read spectrophotometrically at 663 and 645 nm and the calculations were performed using the formula given below:

Chlorophyll a (mg/g FW) = [(12.7 × A663) − (2.6 × A645)] × acetone (mL)/leaf tissue (mg)

Chlorophyll b (mg/g FW) = [(22.9 × A645) − (4.68 × A663)] × acetone (mL)/leaf tissue (mg)

Total chlorophyll content = Chlorophyll a + Chlorophyll b

### 2.6. Estimation of Mineral Content

To estimate the mineral contents, 1 g shade-dried leaves of each of the five varieties was powdered separately and the leaf powder was transferred into separate Erlenmeyer flasks (100 mL capacity) and were digested in triacid containing nitric, perchloric and sulphuric acids (9:3:1 *v*/*v*), for estimation of potassium and phosphorus, and in diacids containing nitric and perchloric acids (9:4), for estimation of calcium, sulphur, nitrogen, sodium, and carbon. Thereafter, 3 g of catalyst mixture [CuSO_4_·6H_2_O (20 g) + Se-powder (1 g) + HgO (3 g) + K_2_SO_4_ (48 g)] plus 10 mL of concentrated sulphuric acid was added to the flasks and incubated overnight. The flasks were placed in the Kjeldahl digestion unit set at 100 °C for 20 min, to digest the mixture followed by adjusting the volume to 100 mL with distilled water. 

#### 2.6.1. Nitrogen

The analysis of nitrogen was done by the Kjeldahl method [27]. An aliquot of 10 mL (of the aforementioned sample) was transferred to a distillation flask (100 mL) and 10 mL of 40% NaOH was added to it. Boric acid was mixed with indicator solution (3 drops) and was kept at the receiving end of the condenser outlet, so that outlet dips in Boric acid. Distillation was carried out by passing the steam into a distillation flask and the color of the solution (boric acid + indicator) changes from reddish-purple to green, and after some time, all the ammonia (NH_3_) was released from the distillation of the sample. After distillation, titration of the sample was carried out against 0.01 N H_2_SO_4_ until the green color changed into purple. A blank (without plant sample) was also used to assess the contamination.

#### 2.6.2. Calcium

Calcium estimation was conducted by titration with EDTA [28]. A 20 mL aliquot of the sample was transferred into a 100 mL Erlenmeyer flask and diluted with 20 mL of distilled water, followed by the addition of sodium hydroxide (2 N, 2 mL), and ammonium purpurate indicator (50 mg). Then, titration was performed with EDTA (0.01 N). The color changed from red to purple. A blank (without plant sample) was also used in the same way.

#### 2.6.3. Carbon

Carbon estimation was carried out by the protocol of Nelson and Sommers [29]. Leaf powder (1 gm) of each variety of *M. oleifera* was transferred into a 500 mL conical flask, containing K_2_Cr_2_O_7_ (1 N, 10 mL), followed by the addition of H_2_SO_4_ (20 mL concentrated) to it. The solution was incubated for 1/2 h followed by the addition of orthophosphoric acid (10 mL), and diphenylamine indicator (1 mL). Finally, the titration was performed against (0.05 N) ferrous ammonium sulphate (139 g of ferrous ammonium sulphate and 15 mL of concentrated H_2_SO_4_).

#### 2.6.4. Sodium and Potassium

The Na and K contents were estimated using a flame photometer, as per the protocol of Gloterman et al. [30] and Sahrawat et al. [31]. Aliquots of MLEs (1 mL) were transferred into the volumetric flasks (50 mL), and a final volume of 50 mL was made with the distilled water. Finally, the solutions were subjected to flame photometer analysis, which was pre-adjusted with the standard solutions (1000 ppm stock solution and 5, 10, 15, 20, and 25 ppm working solution) of sodium (NaCl) and potassium (KCl). Flame photometer readings of standards were plotted to obtain a standard curve. 

#### 2.6.5. Sulphur

Estimation of sulphur was performed by the protocol of Mottershead [32]. MLEs (1 mL each) were transferred into the respective volumetric flasks (50 mL), volumes were made up to 50 mL with distilled water and the transmittance at 470 nm was read spectrophotometrically. Meanwhile, 5, 10, 15, 20, and 25 mL of 100 ppm sulphur standard was prepared in 250 mL volumetric flasks, followed by the addition of buffer salt solution (25 mL) and deionized water to make up the volume to 250 mL. Ten milliliters of solution from each flask was transferred to 50 mL-capacity volumetric flasks, followed by the addition of 1 mL HCl (6 N) and 0.5 g of barium chloride crystals. The flasks were left undisturbed and the final volume was made up to 50 mL with distilled water. Thereafter, the transmittance at 470 nm was recorded using a spectrophotometer and the standard curve was drawn with the transmittance values. 

#### 2.6.6. Phosphorous 

Phosphorous was estimated by the vanadate-molybdate method of Piper [33]. An aliquot of five different MLE samples was transferred to 25 mL volumetric flasks. A few drops of 2,4-dinitrophenol indicator and Na_2_CO_3_ (4 N) were added to it until the yellow color disappeared. The pH was adjusted to 4.8 with HCl (6 N, 2 mL). Thereafter, 5 mL vanadate-molybdate reagent was added and deionized water was added to make up the volume to 25 mL. Transmittance was read at 470 nm after 30 min incubation (as the color starts to develop). Meanwhile, 5, 10, 15, 20, and 25 mL of 100 ppm of the phosphorous standard was transferred to the 25 mL volumetric flasks and the same procedure was followed as mentioned above. Transmittance was read at 470 nm and the standard curve was drawn.

### 2.7. FRAP Assay

For ferric-reducing antioxidant power (FRAP) activity, leaf powder (5 g) of five different varieties of *M. oleifera* were suspended in 100 mL of ethanol in respective conical flasks. Thereafter, the flasks were placed on a rotatory shaker (200 rpm), set at 30 °C for 48 h. The MLE was evaporated in the evaporator shaker and was collected in labeled vials for further use. To the different concentrations of MLE (10–40 µg/mL) of each variety, 625 µL of Na_2_PO_4_ buffer (pH 7.4) plus 625 µL of potassium ferricyanide were added and the mixture was incubated for 20 min at 50 °C. The mixture was allowed to cool and then 625 µL of trichloroacetic acid (TCA) was added. The mixture was centrifuged at 2000 rpm for 10 min. To the supernatant, 625 µL distilled water plus 125 µL FeCl_3_ were mixed, and absorbance was recorded spectrophotometrically at 700 nm [34]. 

### 2.8. HPLC Analysis

High-Performance Liquid Chromatography (HPLC) of the MLEs of five different varieties was performed using an Agilent-1260 system, Germany. The system is equipped with a quaternary pump, VWD UV detector, column C18 (250 L× 4.6 I.D.mm; 5 µm particle size; 100 Å pore size), and an autosampler. The analytes were eluted with a gradient of methanol–0.40% phosphoric acid (ratio 49:51), with a flow rate of 1 mL/min. Estimation of β-sitosterol, quercetin, kaempferol, and moringin contents was performed. Azur 5.0 software was deployed for data analysis [35]. The following formula was used for calculating the bioactive compound % in the sample.

Area of sample/(Weight of sample) × Weight of standard/Area of standard × Dilution of sample/(Dilution of standard) × % Purity of the standard = Bioactive compound % in sample.

### 2.9. 3-(4,5-dimethylthiazol-2-yl)-2,5-diphenyl Tetrazolium Bromide MTT Assay

For MTT assay, the human liver cell line (HepG2) was procured from NCCS, Pune, India, and culture was maintained at Lovely Professional University, Punjab, India. The cells were grown in DMEM media (Gibco, USA), supplemented with fetal bovine serum (10%), in a CO_2_ incubator set at 37 °C. Cytotoxicity evaluation was carried out according to the protocol provided by the American Type Culture Collection (ATCC) [36,37]. Cells were inoculated at a density of 20,000 cells in 96-well plates (Thermo delta surface) and were maintained in a humidified atmosphere [air (64%), CO_2_ (5%)] at 37 °C. These cells were treated with different concentrations (0.25, 0.5, 1, 2, 3, 4, and 5 mg/mL) of MLEs. Thereafter, 50 µL MTT solution [5 mg MTT dye dissolved in 1 mL PBS] was poured into each well and the plate was incubated for 4 h at 37 °C. After incubation, DMSO (15µL) was added into each well. The absorbance was recorded at 570 nm using an ELISA reader. Percentage cytotoxicity was calculated from the absorbance values using the formula given below:

% cell cytotoxicity = 100 − [(O.D. of sample − O.D. of blank)/(O.D. of control − O.D. of blank)] × 100

## 3. Results and Discussion

### 3.1. Total Sugar Content

**Figure 1:**
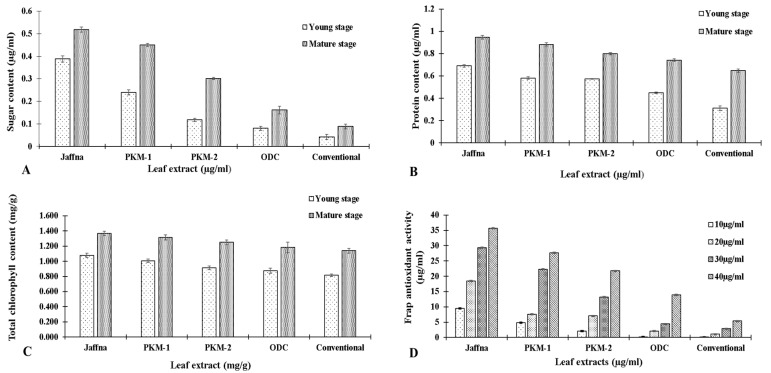
Estimation of biochemical characteristics of five different varieties of *M. oleifera*: (**A**) sugar content, (**B**) protein content, (**C**) total chlorophyll content, and (**D**) FRAP antioxidant activity.

The total sugar content present in MLEs of five *M. oleifera* varieties was analyzed at the young stage (3-months-old) and mature stage (15-months-old). Maximum sugar content was recorded in Jaffna (0.39 ± 0.04 and 0.51 ± 0.01 µg/mL) followed by PKM-1 (0.25 ± 0.001 and 0.45 ± 0.006 µg/mL), PKM-2 (0.12 ± 0.001 and 0.301 ± 0.004 µg/mL), ODC (0.08 ± 0.003 and 0.16 ± 0.01 µg/mL) and Conventional (0.05 ± 0.002 and 0.08 ± 0.01 µg/mL) (Figure 1A). Oxidation-reduction reactions are extremely important in maintaining proper health, by providing an energy source for the body. This process requires catalysts called reducing agents, oxidizing agents, or reducing sugars (glucose, fructose, lactose, and maltose). Sugars (fructose, galactose, glucose, sucrose, lactose, maltose, and raffinose) are the essential component of the human diet [22]. According to Berkovich et al., *M. oleifera* contain reducing sugars [38]. 

### 3.2. Protein Content 

Among all the five varieties, Jaffna showed the highest values of protein content in comparison with the others. The trend observed for the total soluble protein in leaves was: Jaffna (0.695 ± 0.01 and 0.94 ± 0.01 µg/mL) > PKM-1 (0.58 ± 0.01 and 0.88 ± 0.01 µg/mL) > PKM-2 (0.57 ± 0.004 and 0.80 ± 0.01 µg/mL) > ODC (0.44 ± 0.005 and 0.74 ± 0.01µg/mL) > conventional (0.31 ± 0.02 and 0.64 ± 0.01 µg/mL) (Figure 1B). Sanchez-Machado et al. reported the protein content in Moringa plant parts to be 20.66% (immature pods), 30% (mature pods), and 31% (flowers), respectively [39]. In our study, Jaffna was found to be a promising variety that can be used to cope with protein deficiency diseases such as kwashiorkor, marasmus, edema, weak immune system, muscle shrinking, impaired mental health, etc.

### 3.3. Chlorophyll Content 

The chlorophyll molecules are the main pigments which absorb the light in the photosystem reaction centers for photochemical reactions. Chlorophylls are known to have several therapeutic properties (anti-carcinogenic, anti-bacterial, anti-inflammatory, deodorizing, and wound healing activities) [40,41]. In our study *M. oleifera* varieties at two different stages of plant growth (young stage: 3-months-old and mature stage: 15-months-old) were assessed to estimate the amount of chlorophyll a, chlorophyll b and total chlorophyll content. Jaffna variety exhibited the highest chlorophyll content at all the stages. The trend observed was: Jaffna (1.31, 1.18 and 0.82 mg/g) > PKM-1 (1.14, 1.02 and 0.68 mg/g) > PKM-2 (0.96, 0.83 and 0.59 mg/g) > ODC (0.86, 0.70 and 0.49 mg/g) > conventional (0.68, 0.58 and 0.30 mg/g) (Figure 1C). 

### 3.4. Mineral Content

Mineral contents in the five different varieties of *M. oleifera* were estimated by using a flame photometer (Na and K), spectrophotometer (P, and S), Kjeldahl method (N), and titration (Ca and C) as shown in Table 1. Jaffna exhibited the highest mineral content (Na: 10.98%, K: 15.14%, P: 1.11%, S: 1.13%, Ca: 5.9%, N: 2.32% and C: 5.7%) followed by PKM-1, PKM-2, ODC, and conventional. The mineral content in other varieties (PKM-1, PKM-2, ODC, and conventional) were significantly lowered compared with the Jaffna as clearly presented in Table 1. It can be concluded that the Jaffna variety *M. oleifera* is the most nutrient-rich variety, among the five. It contains macro-essential minerals such as Na, K, Ca, N, S, C, and P which make it a potential source of food and is suitable to combat malnutrition. Our results are in agreement with that of Sodamade et al., Oluwole et al., and El Sohaimy et al. [42,43,44]. Jaffna leaves contain a high level of K and Na (15.14 ± 0.1 and 10.98 ± 0.1%), respectively. K works with Na (important electrolytes) and helps in maintaining the water balance and blood pressure. It also prevents heart diseases, osteoporosis, and kidney stones of the body [45]. In the case of plants, K also plays a key role in regulating enzymatic activities; providing strength against drought and stress, and in translocation of photosynthetic products between plant tissues [46]. As per the study of Ghosh et al. [47], our results with regard to the mineral content of *M. oleifera* varieties are also in consonance with the WHO dietary recommendation limits (data not shown).

### 3.5. Frap Antioxidant Activity

The present study demonstrated the antioxidant potential of five different varieties of *Moringa oleifera*, by using FRAP method [48,49]. The results of the study revealed that MLE of Jaffna variety possess high antioxidant property, followed by other four varieties. The trend of the scavenging activity was Jaffna (9.47 µg/mL, 18.48 µg/mL, and 29.39 µg/mL) > PKM-1 (4.82 µg/mL, 7.63 µg/mL, and 22.33 µg/mL) > PKM-2 (2.10 µg/mL, 7.04 µg/mL and 13.18 µg/mL) > ODC (0.17 µg/mL, 2.10 µg/mL, and 4.41µg/mL) > conventional (0.05 µg/mL, 1.08 µg/mL, and 2.86 µg/mL) (Figure 1D). All the samples showed ferric-reducing antioxidant capacity at a concentration dependent manner. Our results are in consonance with the earlier reports which state that flavonoids and phenols are directly responsible for antioxidant activity [35,50,51,52,53].

### 3.6. HPLC Analysis 

**Figure 2:**
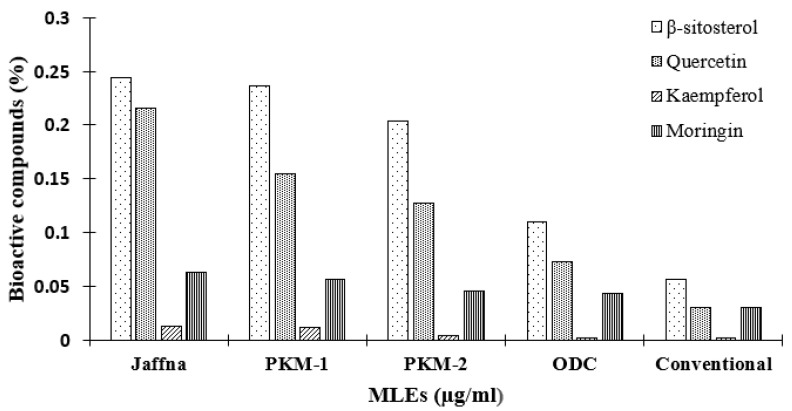
HPLC analysis of the selected anti-cancer compounds reported in MLEs.

To validate these findings, the MLEs of all five varieties of *M. oleifera* were subjected to HPLC for the quantification of four marker bioactive compounds: β-sitosterol, quercetin, kaempferol and moringin. It was found that the Jaffna variety showed the highest β-sitosterol (0.244%), quercetin (0.216%), kaempferol (0.013%), and moringin (0.063%) content, followed by PKM-1 [β-sitosterol (0.236%), quercetin (0.154%), kaempferol (0.012%), and moringin (0.057%)], PKM-2 [β-sitosterol (0.204%), quercetin (0.127%), kaempferol (0.004%), and moringin (0.46%)], ODC [β-sitosterol (0.110%), quercetin (0.073%), kaempferol (0.002%), and moringin (0.43%)], and conventional [β-sitosterol (0.056%), quercetin (0.030%), kaempferol (0.001%), and moringin (0.035%)] (Supplementary Figure S1a–d, Table 2 and Figure 2). 

The present study showed that the different varieties of *M. oleifera* contain sufficient amounts of the aforementioned anti-cancer compounds. The obtained results are in consonance with the findings of Fahey [54], who reported the presence of phytochemicals (zeatin, caffeoylquinic acid, kaempferitrin, isoquercitin, rhamnetin, rhamnose, glucosinolates, and isothiocyanates) in the same plant. Likewise, HPLC and MS analysis performed by Singh et al. [55], showed the presence of chlorogenic acid, vanillin, gallic acid, ferulic acid, and ellagic acid in seeds, fruits, and leaves of *M. oleifera*. Farooq Anwar et al. have also reported that the leaves of M. oleifera contain unique compounds such as o-coumaric acid, epicatechin, niazirinin, niaziminin A, niazirin, 4-[(4′-O-acetyl-L rhamnosyloxy) benzyl] isothiocyanate, 3-caffeoylquinic, and 5-caffeoylquinic acid [56]. A non-exhaustive list of phytochemicals reported in *M. oleifera* is shown in Supplementary Table S3. The comparison between the five varieties of *M. oleifera* indicates that the Jaffna variety is endowed with a higher content of anti-cancer compounds than the other four. Thus, these results suggest the cultivation and consumption of Jaffna leaves in cancer-affected zones worldwide.

### 3.7. MTT Assay

**Figure 3:**
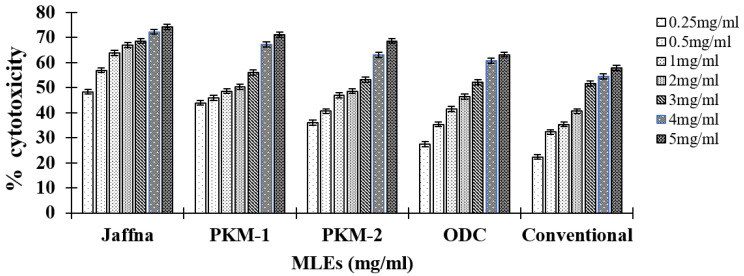
Graphical representation of MTT assay using MLEs.

MLEs of all five varieties were also evaluated for their cytotoxic activity against the HepG2 cancer cell line, through the MTT assay. Figure 2 reveals the percentage (%) cytotoxicity of different concentrations of the MLEs (0.25, 0.5, 1, 2, 3, 4, and 5 mg/mL) of five varieties. MLEs of all varieties exhibited dose-dependent cytotoxic activity against the cancer cell line, which were found to be significant. 5-Fluorouracil was used as a standard (0.5, 0.1, 0.2, 0.3, 0.4, 0.5 and 0.6 mM). The IC50 value of 5-FU was 0.87 mM/mL. The trend observed for cytotoxicity on the basis of IC50 values was: conventional (1.22 mg/mL) > ODC (0.90 mg/mL) > PKM-2 (0.65 mg/mL) > PKM-1 (0.35 mg/mL) > Jaffna (0.15 mg/mL). Jaffna leaf extract showed a strong cytotoxic activity as compared to the other four varieties (Table 3 and Figure 3). Thus, it can be used as a novel alternative and complementary therapeutic agent against cancer treatment regimes.

Cancer cells possess tremendous potential to divide and therefore anticancer agents are required to stop their growth by targeting them. Interestingly, MLE performs a wide range of biological activities and even targets several proteins and biomolecules to retard cancer progression [57,58]. It contains glucosinolates that have the ability to induce apoptosis and are very effective against cancer [59]. Berkovich et al. reported that MLE inhibits pancreatic cancer cell growth [38]; Thorneloe et al.—breast cancer cell growth [60] and Sadek et al. [11]—liver cancer (induced by diethylnitrosamine in rats). *M. oleifera* also inhibits the cell viability of liver carcinoma, lymphoblastic, and myeloid leukemia [61]. These activities of Moringa can be attributed to the presence of different classes of anticancer compounds such as kaempferol, niazimicin, β-sitosterol-3-O-β-D-glucopyranoside, and 4-(α-L-rhamnosyloxy) benzyl isothiocyanate [59]. These target the cell cycle by accumulating the cells at the sub-G1 phase. According to Tiloke et al., hot water MLE exhibits antiproliferative activity against A549 lung cancer cells [57]. The reason behind this is that it increases reactive oxygen species (ROS), which leads to the induction of caspases, cleavage of PARP-1, and P53 initiates apoptosis of cancer cells. MLE up-regulates apoptotic markers which leads to the death of cancer cells and down-regulates the NFKB pathway which further decreases the expression of P-IKB α, IKB α, and P65 proteins. Together, these induce cytotoxicity in cancer cells [62]. The present study reports the cytotoxic potential of five different varieties of *M. oleifera* on the HepG2 cell line. Jaffna variety showed the highest cytotoxic effect on cancer the cell line as compared to the other varieties.

## 4. Conclusions

The easiest solution for affordable health and nutrition is to shake hands with nature. As mentioned, mother nature has provided mankind with the gift of ‘superfood trees’ (known as *M. oleifera*) to combat malnutrition. The fact that Moringa is a source of nutrition and medicine has been tested over time and is deeply rooted in Ayurveda, Siddha, Unani, and the traditional Chinese system of medicine (TCM). It is interesting to note that people of all age groups can consume moringa leaf powder and can replenish vitamins and minerals. The present study shows the comparison of biochemical properties of PKM-1, PKM-2, ODC, Jaffna, and conventional varieties of *M. oleifera* on the basis of different parameters: Frap antioxidant activity, sugar, protein and mineral contents, percentage of phytochemicals (four anticancer compounds), and cytotoxicity against the HepG2 cell line. By assessing these parameters, it was possible to conclude that the Jaffna variety is the most beneficial variety in terms of nutrition, anticancer bioactive compounds, and cytotoxic activity. However, the Jaffna variety needs to be explored further to be involved in reforestation programs so that many people may fully capitalize on its amazing benefits. Thus, meticulous studies on other nutritional and medicinal parameters of Jaffna should be conducted to explore its potential as an alternative and cost-effective source of nutrition and medicine, for better healthcare.

## Figures and Tables

**Table 1:** Percent mineral content of five *Moringa oleifera* varieties.

Mineral	Jaffna (a)	PKM-1 (b)	PKM-2 (c)	ODC (d)	Conventional (e)
K	15.14 ± 1.1	11.07 ± 1 a *	8.50 ± 1 a ** b *	6.77 ± 1 a ** b *	4.94 ± 0.5 a ** b **c *
Na	10.98 ± 1	9.77 ± 1	8.90 ± 1	7.70 ± 1 a *	6.61 ± 1 a * b *
Ca	5.90 ± 0.2	4.96 ± 0.2 a *	3.96 ± 0.3 a ** b **	3.02 ± 0.1 a * b **c *	2.32 ± 0.1 b ** c **d *
C	5.70 ± 0.1	4.99 ± 0.1 a **	4.17 ± 0.1 a * b **	3.79 ± 0.1 b ** c *	2.89 ± 0.1 c ** d **
N	2.32 ± 0.1	2.08 ± 0.1	1.68 ± 0.1 a ** b *	1.34 ± 0.1 a ** b ** c *	0.99 ± 0.1 a ** b ** c ** d *
P	1.11 ± 0.08	0.86 ± 0.02 a **	0.78 ± 0.05 a **	0.63 ± 0.04 a ** b *c *	0.51 ± 0.02 a ** b ** c **
S	1.13 ± 0.09	0.84 ± 0.03 a *	0.76 ± 0.05 a **	0.58 ± 0.06 a ** b*	0.43 ± 0.06 a ** b ** c **

Data were analyzed using one way ANOVA and Tukey’s HSD post-hoc test. Levels of significance were represented in the form of (b–e) *p* < 0.001 (**), (b–e) *p* < 0.05 (*) when compared with Jaffna (a) group.

**Table 2:** HPLC analysis of selected compounds reported in MLEs.

Variety	Quantified Compound							
	β-Sitosterol		Quercetin		Kaempferol		Moringin	
	Area	% Content	Area	% Content	Area	% Content	Area	% Content
Jaffna	154,084	0.244	355,765	0.216	663,751	0.013	1,254,800	0.063
PKM-1	149,743	0.236	234,489	0.155	652,556	0.012	1,133,682	0.057
PKM-2	129,271	0.204	204,488	0.127	239,864	0.004	898,404	0.046
ODC	72,338	0.110	118,729	0.073	111,264	0.002	864,931	0.043
Conventional	35,945	0.056	94,796	0.030	71,290	0.001	671,978	0.03

**Table 3:** MTT assay using MLEs of five varieties of *Moringa oleifera*.

Varieties	Cytotoxicity at Different Concentrations (mg/mL)								
	0.25	0.5	1	2	3	4	5	*p* Value	IC50 (mg/mL)
Jaffna	48.37 ± 0.001	56.97 ± 0.002	63.86 ± 0.001	67.01 ± 0.003	68.64 ± 0.009	72.27 ± 0.008	74.28 ± 0.021	*p* < 0.001	0.15
PKM-1	43.97 ± 0.001	45.94 ± 0.003	48.66 ± 0.004	50.38 ± 0.004	60.2 ± 0.007	67.20 ± 0.004	71.22 ± 0.004	*p* < 0.001	0.35
PKM-2	36.13 ± 0.046	40.63 ± 0.002	46.94 ± 0.004	48.66 ± 0.004	53.25 ± 0.003	63.09 ± 0.001	68.64 ± 0.025	*p* < 0.001	0.65
ODC	27.53 ± 0.005	35.37 ± 0.007	41.49 ± 0.001	46.36 ± 0.004	52.10 ± 0.001	60.80 ± 0.003	63.19 ± 0.008	*p* < 0.001	0.90
Conventional	22.37 ± 0.014	32.31 ± 0.009	35.37 ± 0.007	40.63 ± 0.002	51.62 ± 0.008	54.58 ± 0.002	57.83 ± 0.002	*p* < 0.001	1.22

IC50 value of 5-FU (standard): 0.87 mM/mL. Data were analyzed using one-way ANOVA, Levels of significance were represented in the form of *p* < 0.001 when compared with and between groups.

## Data Availability

The data presented in this study are available upon request from the corresponding author.

## Supplementary Materials

The following are available online at https://www.mdpi.com/article/10.3390/plants10112348/s1, Figure S1: (a) HPLC chromatogram for estimation of β-sitosterol content in *M.oleifera* varieties leaf extracts. [A] standard compound [B] Jaffna [C] PKM-1 [D] PKM-2 [F] ODC and [F] Conventional. (b) HPLC chromatogram for estimation of Quercitin content in *M. oleifera* varieties leaf extracts. [A] Standard compound [B] Jaffna, [C] PKM-1 [D] PKM-2 [F] ODC and [F] Conventional. (c) HPLC chromatogram for estimation of Kaempferol content in *M.oleifera* varieties leaf extracts. [A] Standard compound [B] Jaffna [C] PKM-1, [D] PKM-2 [F] ODC and [F] Conventional. (d) HPLC chromatogram for estimation of Moringin content in *M. oleifera* varieties leaf extracts. [A] Standard compound [B] Jaffna [C] PKM-1 [D] PKM-2 [F] ODC and [F] Conventional, Table S1: Anti-bacterial activity (bacterial zone of inhibition values) of *M. oleifera* varieties, Table S2: Plant growth promoting activity of *M. oleifera* varieties leaf extract on *S. rebaudiana*, Table S3: Phytochemical constituents reported in different parts of *Moringa oleifera*.
