# Supplementary material for: Phytochemical Analyses, Antioxidant and Anticancer Activities of Ethanolic Leaf Extracts of Moringa oleifera Lam. Varieties
Source: Plants (Basel). 2021 Oct 30;10(11):2348. doi: 10.3390/plants10112348 (PMC8625309; doi:10.3390/plants10112348)
Supplement: Supplementary file 1 [file plants-10-02348-s001.zip › plants-1412539-supplementary.pdf]

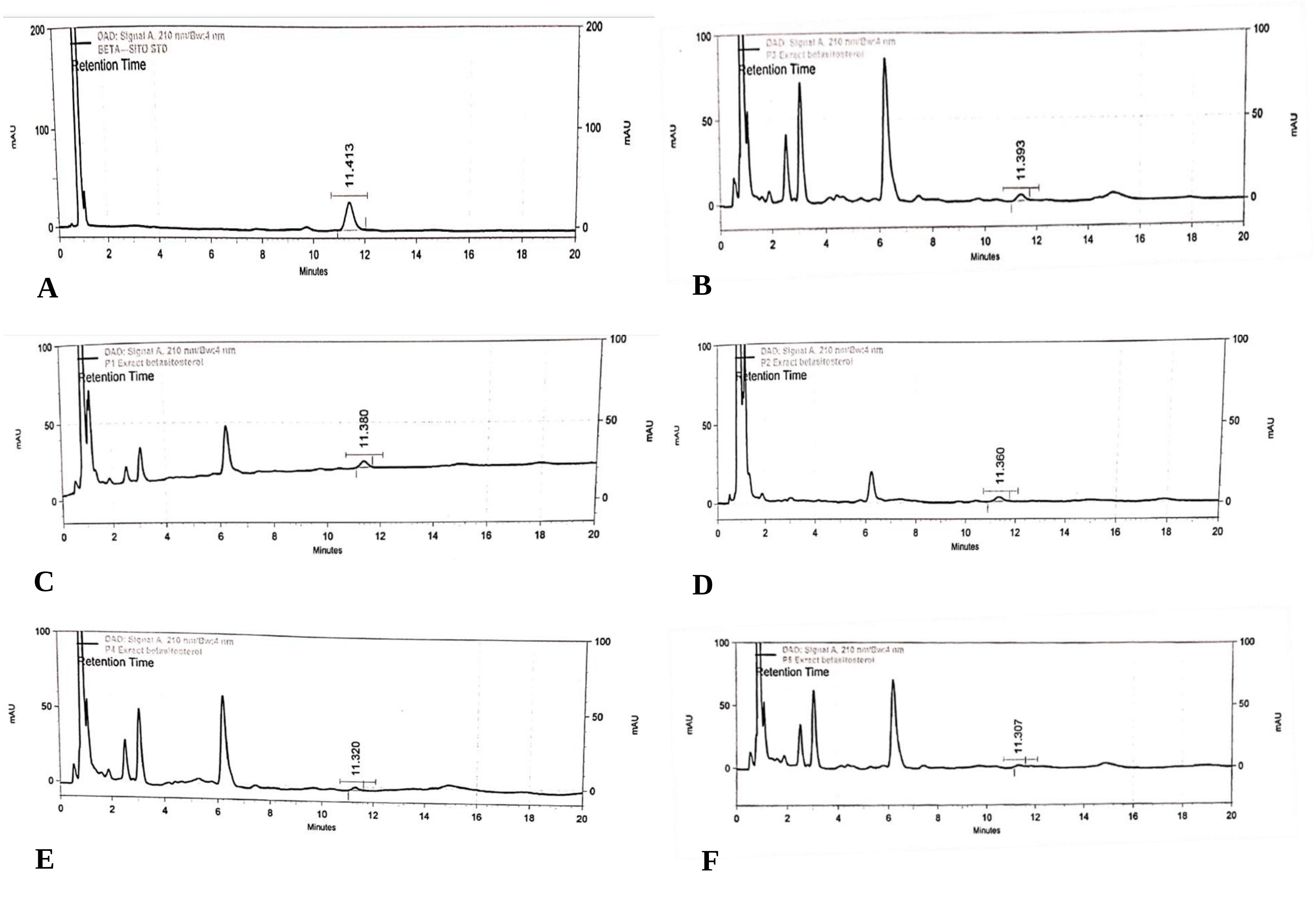

Figure S1a: HPLC chromatogram for estimation of β-sitosterol content in *M.oleifera* varieties leaf extracts.[A] standard compound [B] Jaffna [C] PKM-1 [D] PKM-2 [F] ODC and [F] Conventional..

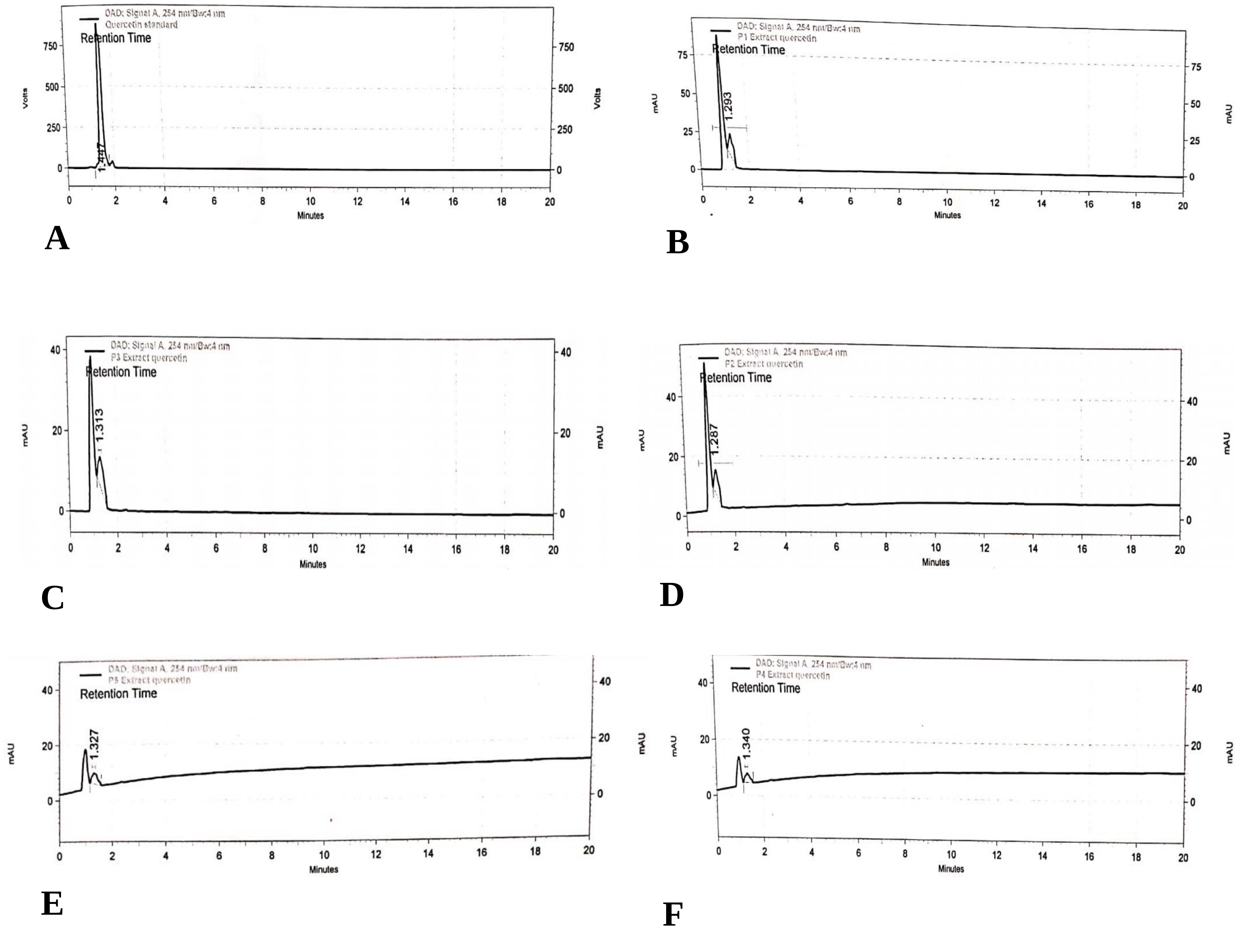

Figure S1b: HPLC chromatogram for estimation of Quercitin content in *M.oleifera* varieties leaf extracts. **[A]** Standard compound **[B]** Jaffna, **[C]** PKM-1 **[D]** PKM-2 **[F]** ODC and **[F]** Conventional.

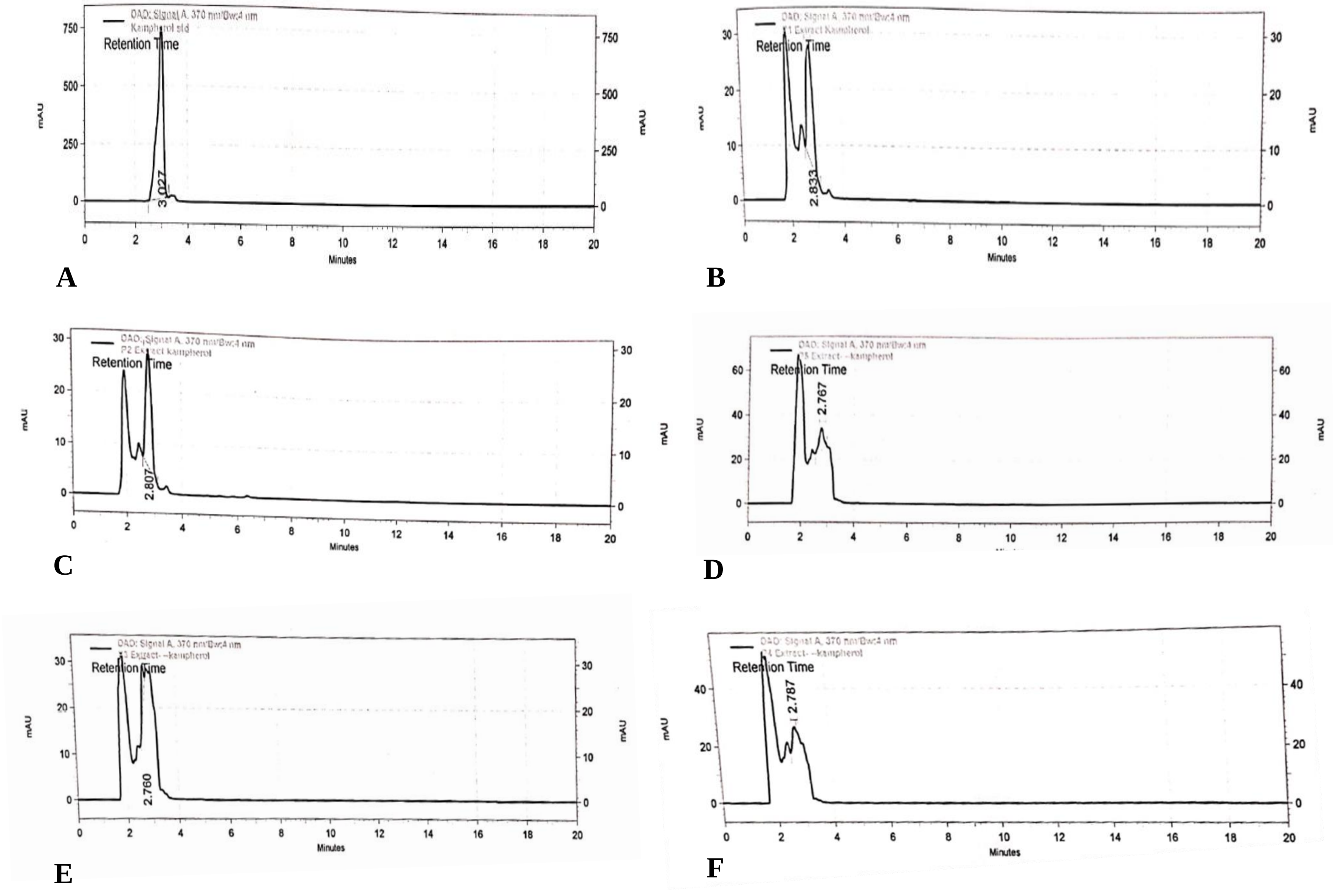

Figure S1c : HPLC chromatogram for estimation of Kaempferol content in *M.oleifera* varieties leaf extracts.**[A]** Standard compound **[B]** Jaffna **[C]** PKM-1, **[D]** PKM-2 **[F]** ODC and **[F]** Conventional.

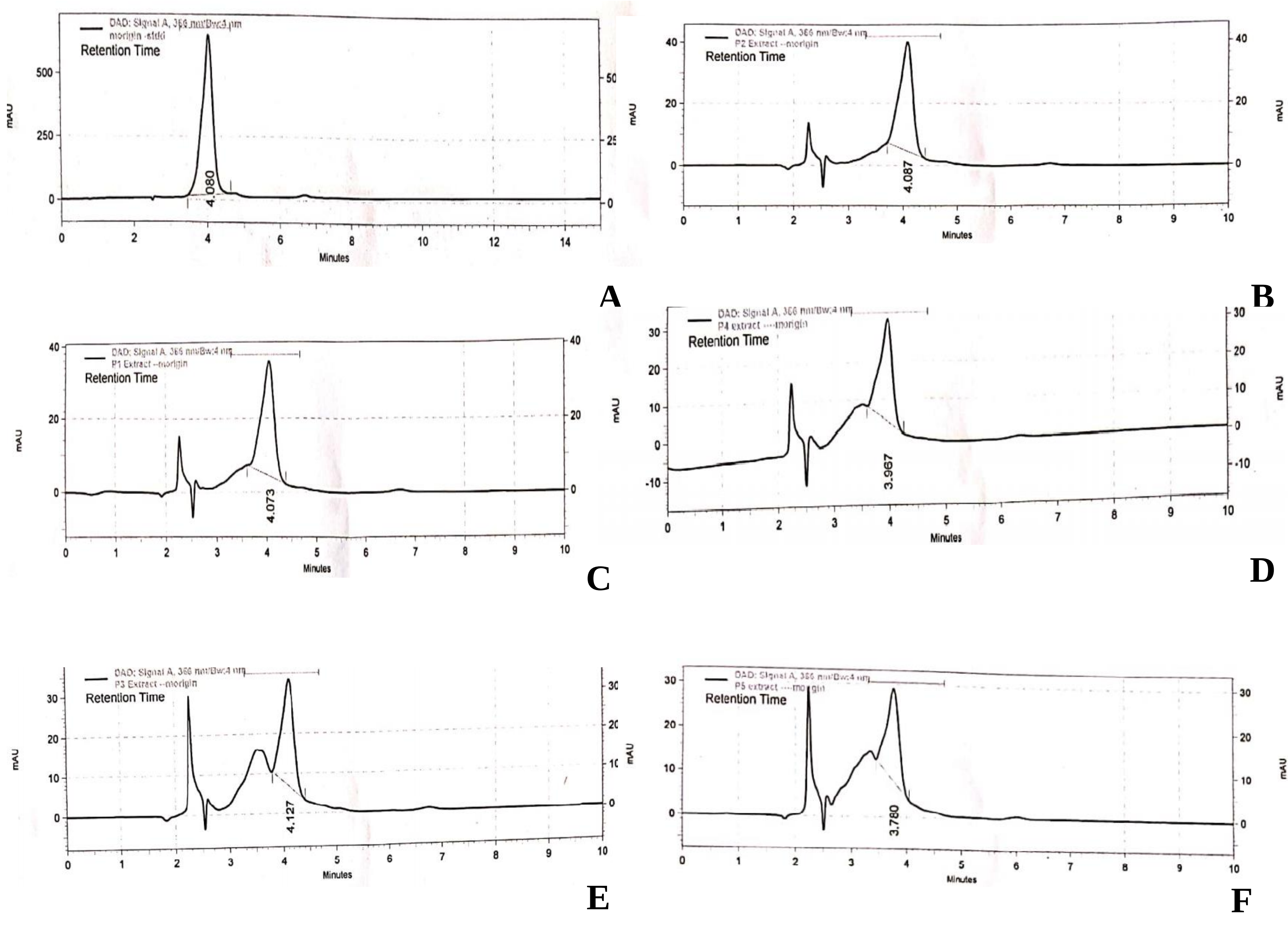

Figure S1d: HPLC chromatogram for estimation of Moringin content in *M.oleifera* varieties leaf extracts. **[A]** Standard compound **[B]** Jaffna **[C]** PKM-1 **[D]** PKM-2 **[F]** ODC and **[F]** Conventional.

Supplementary Table S1: Anti-bacterial activity (bacterial zone of inhibition values) of *M. oleifera* varieties.

	**Aqueous leaf extract**				**Aqueous seed extract**			
**Varieties**	***E. coli***	***B. subtilis***	***P. aeruginosa***	***S. aureus***	***E. coli***	***B. subtilis***	***P. aeruginosa***	***S. aureus***
Jaffna	13.63±0.65^{a}	14.66±0.41^{a}	16.83±0.20^{a}	11.83±0.15^{a}	12.53±0.45^{a}	14.86±0.15^{a}	16.66±0.35^{a}	10.63±0.30^{a}
PKM-1	10.13±0.61^{b}	12.66±0.41^{b}	15.83±0.20^{b}	10.70±0.26^{b}	10.66±0.35^{b}	12.70±0.30^{b}	15.73±0.30^{b}	9.66±0.30^{b}
PKM-2	9.76±0.25^{c}	11.20±0.20^{c}	13.80±0.20^{c}	13.36±3.78^{c}	8.63±0.40^{c}	10.56±0.40^{c}	13.63±0.47^{c}	7.70±0.30^{c}
ODC	8.60±0.45^{d}	13.83±0.41^{d}	13.63±0.35^{d}	8.86±0.85^{d}	8.63±0.47^{d}	9.33±0.20^{d}	11.80±0.35^{d}	10.60±0.36^{d}
Conventional	6.76±0.32^{e}	10.03±0.90^{e}	9.96±0.90^{e}	5.73±0.30^{e}	6.93±0.50^{e}	8.56±0.45^{e}	9.70±0.26^{e}	3.53±0.26^{e}
	**Ethanolic leaf extract**				**Ethanolic seed extract**			
Jaffna	23.6±0.36^{a}	26.00±0.80^{a}	27.80±0.43^{a*}	25.00±0.40^{a}	22.56±0.45^{a}	23.80±0.20^{a}	25.86±0.15^{a}	20.66±0.30^{a}
PKM-1	22.4±0.50^{b}	24.90±0.45^{b}	23.90±0.26^{b}	23.00±0.10^{b}	20.06±0.81^{b}	20.20±0.20^{b}	24.63±0.35^{b}	19.83±0.15^{b}
PKM-2	19.83±0.85^{c}	20.33±0.61^{c}	23.23±0.20^{c}	19.83±0.15^{c}	17.70±0.36^{c}	20.13±0.51^{c}	21.73±0.25^{c}	18.26±0.30^{c}
ODC	18.0±0.40^{d}	20.83±0.20^{d}	20.26±0.30^{d}	16.73±0.25^{d}	12.70±0.20^{d}	13.66±0.41^{d}	18.60±0.36^{d}	9.70±0.30^{d}
Conventional	13.66±0.41^{e}	18.46±0.61^{e}	18.43±0.40^{e}	14.63±0.40^{e}	9.50±0.40^{e}	14.70±0.43^{e}	16.70±0.30^{e}	8.70±0.25^{e}

*LSD: least significantly different at $p \leq 0.05$ according to the posthoc test. Each value is presented as mean of triplicate treatments.

Supplementary Table S2: Plant growth promoting activity of *M. oleifera* varieties leaf extract on *S. rebaudiana.*

Foliar spray	Shoot length (cm)	Root length (cm)	No. of leaves	Plant dry weight (gm)	Chlorophyll a content (mg/g FW)	Chlorophyll b content (mg/g FW)	Carotenoid content (mg/g FW)	Total soluble sugars content (mg/g FW)	Total protein content (mg/g FW)	Total Phenolic content (µg/g FW)	Total flavonoid content (µg/g FW)
Water	12.46±0.40[a*]	6.05 ± 0.30[a]	48±3.05[a]	1.32 ± 0.01[a]	0.75 ± 0.03[a]	0.78 ± 0.02[a]	0.25 ± 0.02[a]	9.31± 0.78[a]	4.40 ± 0.62[a]	230.41 ± 0.25[a]	112.21 ± 0.66[a]
Jaffna	35.43 ±0.20[b]	30.70 ±0.50[b]	138±3.50[b]	4.90 ± 0.43[b]	0.85 ± 0.03[b]	0.87 ± 0.01[b]	0.45 ± 0.01[b]	25.69 ± 0.63[b]	8.43 ± 0.84[b]	420.46 ± 0.50[b]	194.43 ± 0.25[b]
PKM-1	33.26 ±0.30[c]	22.40 ±0.30[c]	122±3.60[c]	4.46 ± 0.34[c]	0.83 ± 0.01[c]	0.86 ± 0.01[c]	0.41 ± 0.01[c]	22.80± 0.82[c]	7.75 ± 0.49[c]	387.20 ± 0.20[c]	185.50± 0.36[c]
PKM-2	28.40 ±0.30[d]	25.50 ±0.20[d]	103±4.30[d]	4.01 ± 0.20[d]	0.81 ± 0.01[d]	0.83 ± 0.01[d]	0.35 ± 0.01[d]	17.84 ± 0.84[d]	7.28 ± 0.78[d]	356.36 ± 0.32[d]	162.13 ± 0.90[d]
ODC	27.10 ±0.26[e]	15.70 ±0.30[e]	88 ± 3.50[e]	3.13 ± 0.48[e]	0.79 ± 0.01[e]	0.82 ± 0.01[e]	0.30 ± 0.02[e]	14.83 ± 0.79[e]	6.69 ± 0.53[e]	308.36 ± 0.15[e]	135.30 ± 0.26[e]
Conventional	20.63 ±0.20[f]	12.50 ±0.90[f]	64 ± 4.04[f]	2.82 ± 0.04[f]	0.78 ± 0.01[f]	0.80 ± 0.01[f]	0.27 ± 0.02[f]	12.05 ± 0.81[f]	5.66 ± 0.55[f]	280.40 ± 0.40[f]	120.86 ± 0.90[f]

*LSD: least significantly different at $p \leq 0.05$ according to the posthoc test. Each value is presented as mean of triplicate treatment

Supplementary Table S3: Phytochemical constituents reported in different parts of *Moringa oleifera.*

Plant part	Bioactive compound	Biological activity	Chemical structure	Reference(s)
Leaves	Niazirin	Hypotensive activity	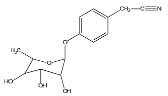	[1,2–5]
	Niazirinin		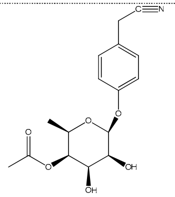	
	4-(4′-O-acetyl-α-Lrhamnopyranosyloxy) benzyl isothiocyanate	Antibacterial, Antitumor, Anticancer, Antiulcer, Antispasmodic, Antihyperthyroidic, Estrogenic, Hypoglycaemic, Hypocholesterolemic,Purgative and Abortifacient properties	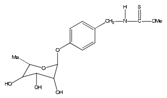	
	Niaziminin A and B	Antitumor and Hypotensive activity	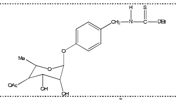	
	Niazimicin	Antispasmodic, Anticancer, Antibacterial, Antitumour	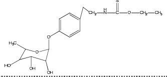	
Bark	4-(α Lrhamnopyranosyloxy)benzyl glucosinolate	Hypotensive, Anticancer, Antibacterial, Antifungal activity	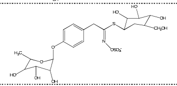	[1]
Stem	β-sitosterol	Anticancer and hypotensive activity	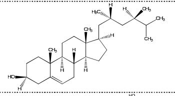	[1,4,6,7]
	4-(α-Lrhamnopyranosyloxy)benzyl glucosinolate	Hypotensive, Anticancer, Antibacterial and Antifungal activity	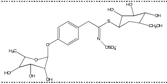	
Roots	Moringine	Cardiac stimulant, hypolipidimic bronchodilator activity	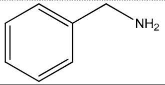	

	Pterygospermin	Antimicrobial, antibacterial and Anti-fungal properties	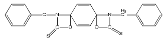	
Flower	Quercetin	Antioxidant and Hepatoprotective activity	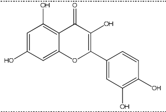	[1,7]
	Isoquercitin	Antioxidant activity	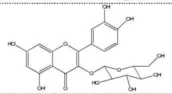	
	Kaempherol	Antioxidant activity	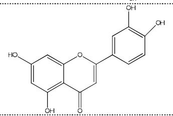	
	Kaempferitrin	Antioxidant, Antimicrobial and Anti-inflammatory activity	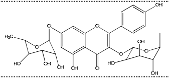	
	Ascorbic acid	Antioxidant activity	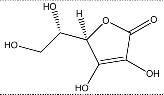	
	O-ethyl-4-(α-Lrhamnosyloxy)benzyl carbamate	Antitumor activity	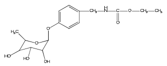	
Pods/Seeds	4(α- L-rhamnosyloxy)-benzyl isothiocyanate	Antitumor, Antimicrobial, Hypotensive, Anticancer and Antibacterial activity	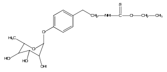	[1,4]
	Niazimicin	Antispasmodic, Anticancer and Antitumour	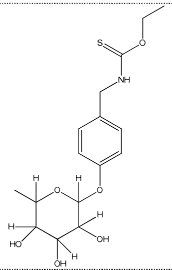	
	3-O-(6′-O-oleoyl-β-Dglucopyranosyl)-β-sitosterol	Antitumor and Anticancer property	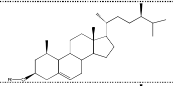	
	β-sitosterol-3-O-β-Dglucopyranoside	Anticancer property	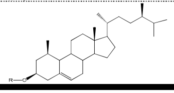	

	Niazirin	Hypotensive activity	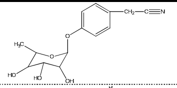	
	β-sitosterol	Anticancer property	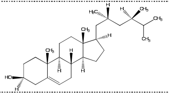	
	Glycerol-1-(9octadecanoate)	Anticancer property	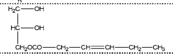	
	4-(α-Lrhamnopyranosyloxy) benzyl glucosinolate	Hypotensive, Anticancer, Antibacterial and Antifungal activity	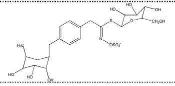	
	β-carotene.	Antioxidant activity	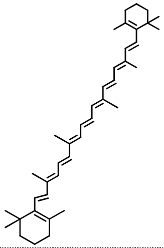	
	Vitamin A	Prevents blindness, maternal mortality, enhances lactation (breast milk); Enhances immunity to fight infections	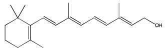	

1. Anwar, F.; Latif, S.; Ashraf, M.; Gilani, A.H. *Moringa oleifera*: A food plant with multiple medicinal uses. *Phytother. Res.* **2007**, *21*, 17–25.
2. Gilani, A.H.; Aftab, K.; Suria, A.; Siddiqui, S.; Salem, R.; Siddiqui, B.S.; Faizi, S. Pharmacological studies on hypotensive and spasmolytic activities of pure compounds from *Moringa oleifera*. *Phytother. Res.* **1994**, *8*, 87–91.
3. Bennett, R.N.; Mellon, F.A.; Foidl, N.; Pratt, J.H.; Dupont, M.S.; Perkins, L.; Kroon, P.A. Profiling glucosinolates and phenolics in vegetative and reproductive tissues of the multi-purpose trees *Moringa oleifera* L.(horseradish tree) and *Moringa stenopetala* L. *J. Agric. Food Chem.* **2003**, *51*, 3546–3553.
4. Kumar, P.S.; Mishra, D.; Ghosh, G.; Panda, C.S. Medicinal uses and pharmacological properties of *Moringa oleifera*. *Int. J. Phytomed.* **2010**, *2*, 210–216.
5. Farooq, F.; Rai, M.; Tiwari, A.; Khan, A.A.; Farooq, S. Medicinal properties of *Moringa oleifera*: An overview of promising healer. *J. Med. Plants Res.* **2012**, *6*, 4368–4374.
6. Sutherland, J.; Folkard, G.; Grant, W. Natural coagulants for appropriate water treatment: A novel approach. *Waterlines* **1990**, *8*, 30–32.
7. Ruckmani, K.; Kavimani, S.; An, R.; Jaykar, B. Effect of *Moringa oleifera* Lam on paracetamol-induced hepatotoxicity. *Indian J. Pharm. Sci.* **1998**, *60*, 33.
